# Correlative study of epigenetic regulation of tumor microenvironment in spindle cell melanomas and cutaneous malignant peripheral nerve sheath tumors

**DOI:** 10.1038/s41598-020-69787-1

**Published:** 2020-08-03

**Authors:** Theodore Vougiouklakis, Phyu P. Aung, Varshini Vasudevaraja, Victor G. Prieto, Carlos A. Torres-Cabala, Erik P. Sulman, Matija Snuderl, George Jour

**Affiliations:** 1 Department of Pathology, NYU Langone Health, NYU Grossman School of Medicine, 550 First Ave, New York, NY 10016 USA; 2 Department of Pathology, Section of Dermatopathology, The University of Texas MD Anderson Cancer Center, Houston, TX USA; 3 Department of Applied Bioinformatics Laboratories, NYU Langone Health, NYU Grossman School of Medicine, New York, NY USA; 4 Department of Radiation Oncology, NYU Langone Health, NYU Grossman School of Medicine, New York, NY USA; 5 Laura and Isaac Perlmutter Cancer Center, NYU Langone Health, New York, NY USA; 6 Department of Dermatology, NYU Langone Health, NYU Grossman School of Medicine, New York, NY USA

**Keywords:** Cancer, Epigenetics, Gene expression

## Abstract

The tumor microenvironment (TME) plays critical roles in tumor growth and progression, however key regulators of gene expression in the TME of cutaneous malignant peripheral nerve sheath tumor (C-MPNST) and spindle cell melanoma (SCM) have not been well elucidated. Herein, we investigate the epigenetic regulation of promoters and gene bodies and their effect on the TME composition of C-MPNSTs and SCMs. A cohort of 30 patients was analyzed using differential gene expression (DGE) and gene set enrichment analysis (GSEA) using the Nanostring platform. Methylation analysis was carried out utilizing an Infinium Methylation EPIC array targeting 866,562 methylation site (CpG) islands. DGE revealed overexpression of genes related to mast cells in the TME of SCMs, and a predominance of exhausted CD8^+^ T cells and macrophages in the TME of C-MPNSTs. Interestingly, we further observed promoter hypermethylation in key overexpressed genes and corresponding gene body hypomethylation. Analysis using ENCODE ChIP-sequencing data identified *CTCF* as the common transcription factor at the site of the hypomethylated probe. These findings support that the TME composition of C-MPNSTs and SCMs is at least partially independent on promoter methylation status, suggesting a possible relationship between gene body enhancers and expression of key TME genes in both entities.

## Introduction

Significant histomorphologic challenges frequently arise in the distinction between cutaneous malignant peripheral nerve sheath tumor (C-MPNST) and spindle cell melanoma (SCM) on account that both entities display immunopositivity for S100 and typically do not exhibit immunoreactivity for more specific markers of melanocytic differentiation^1–3^. Morphologic infidelity further presents diagnostic hurdles in discriminating between C-MPNSTs and certain amelanotic SCMs, particularly in sun-damaged areas of the skin^2^. Rendering the correct diagnosis is crucial as prognosis and patient management are different among these lesions.

MPNSTs constitute approximately 3–10% of all soft tissue sarcomas and portend dismal prognosis with propensity for local invasion^4,5^. These neoplasms have the proclivity to arise from the deep soft tissues of the upper and lower limbs, and from the trunk^6^. C-MPNSTs represent a rare subtype localized to the dermis and/or subcutis with unusual clinical presentation arising primarily in association with neurofibromas^7^. The mainstay of treatment is complete surgical excision with wide margins. SCMs are variants of melanoma that display fascicles of spindle cells arranged in a sheet-like manner with < 90% desmoplastic stroma in chronically sun-damaged skin. While conventional *BRAF* and *NRAS* mutations are not highly prevalent in SCMs, more recently, alterations in genes involved in aberrant activation of the mitogen-activated protein kinase (MAPK) and phosphoinositide 3-kinase (PI3K) signaling pathways have been identified with possibility for targeted therapies with small molecule inhibitors^8^.

The tumor microenvironment (TME) establishes an intricate niche whereby tumor cells interact with neighboring stromal and extracellular matrix elements to create a collaborative setting that promotes tumor progression and an aptitude for metastasis^9,10^. Exploration of these entities has provided dynamic insights into the molecular classification and methylome signatures that aid in distinguishing between C-MPNSTs and SCMs, while further laying the foundation for potential future targeted therapies^11,12^. Recently, our group identified pivotal immune pathway perturbations in the TME detected in both C-MPNSTs and SCMs that included the janus kinase-signal transducer and activator of transcription (JAK/STAT) pathway, nuclear factor-κB (NF-κB), and CXCL12-CXCR4, in addition to *CD274* (PD-L1) and *CTLA4* overexpression^11^. Furthermore, subsequent interrogation of genomic methylation profiles identified a distinct methylome signature implicating the promoter region of the Branched-Chain Aminotransferase 1 (*BCAT1*) gene. Hypomethylation of the *BCAT1* promoter region was seen in the C-MPNST group but not in SCMs, resulting in transcriptional activation and significant enrichment in gastric and breast cancer-related genes as emphasized on functional genomic pathway analysis^12^. Yet to date, the epigenetic regulation of the TME and its key players have not been well investigated in C-MPNSTs and SCMs.

Herein, we seek to investigate the epigenetic mechanisms governing the TME composition of C-MPNSTs and SCMs in an attempt to identify specific epigenetic events that would explain, at least partially, the difference in clinical behavior and provide actionable targets that warrant investigation in future studies. This was pursued implementing a tri-dimensional approach integrating differential gene expression (DGE), tandem genome-wide methylation with differential methylation analysis, and gene set enrichment analysis (GSEA). Finally, we employed a methylation driven approach to estimate the TME composition in both entities (MethylCIBERSORT) and correlate its findings with the DGE and GSEA results.

## Results

### Case selection

**Figure 1:**
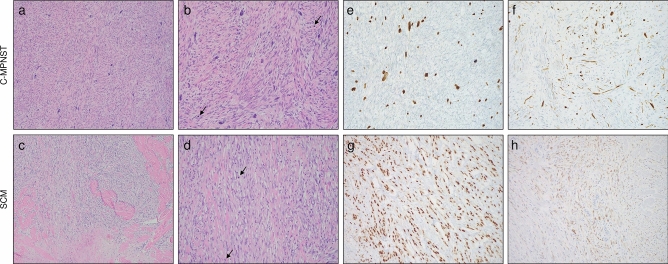
Histomorphology of C-MPNST and SCM cases in the cohort. (**a**,**b**) Representative photomicrographs of C-MPNST. (**a**) Lower power magnification showing a very cellular spindle cell neoplasm growing in fascicles (10× Objective Magnification). (**b**) Higher power magnification of the C-MPNST showing highly atypical spindle cells with mitotic activity depicted by the arrows (20× Objective Magnification). (**c**,**d**) Representative photomicrographs of SCM. (**c**) Tumor showing spindle cell morphology growing in fascicles with intervening sclerotic stroma invading in the subcutaneous tissue with associated lymphocytic infiltrate at the tumor edges (10× Objective Magnification). (**d**) Higher power magnification showing spindle cells with hyperchromatic nuclei and prominent cytologic atypia (20× Objective Magnification). Mitotic figures are noted too (depicted by the arrows). Immunostains for SOX10 and S100 are shown for C-MPNST (**e**,**f**) and SCM (**g**,**h**), respectively (20× Objective Magnification). Note the diffuse pattern of both SOX10 (nuclear) and S100 (nuclear and cytoplasmic) immunoreactivity in SCM, and the patchy SOX10 (nuclear) and S100 (nuclear and cytoplasmic) immunoreactivity in C-MPNST.

Our cohort is comprised of 30 patients with either C-MPNST (*n* = 15) or SCM (*n* = 15; mixed type) with representative photomicrographs shown in Fig. 1a–d. No significant difference in age (C-MPNST [mean = 56 years] vs. SCM [mean = 62 years]; *p* = 0.3, Student's *t*-test) was identified among the patient cohort, whereas a male predominance was noted in both groups (73% in C-MPNST vs. 80% in SCM). All cases were reviewed and confirmed by two different experienced dermatopathologists and soft tissue pathologists in order to reach a final diagnosis. The workup included ancillary immunohistochemical studies with SOX10 and S100 for both C-MPNST (Fig. 1e,f) and SCM (Fig. 1g,h), respectively, in addition to the identification of dermal and subcutaneous plexiform and/or diffuse neurofibromas for C-MPNST.

### Differential gene expression in C-MPNST and SCM reveals distinct TME composition

**Figure 2:**
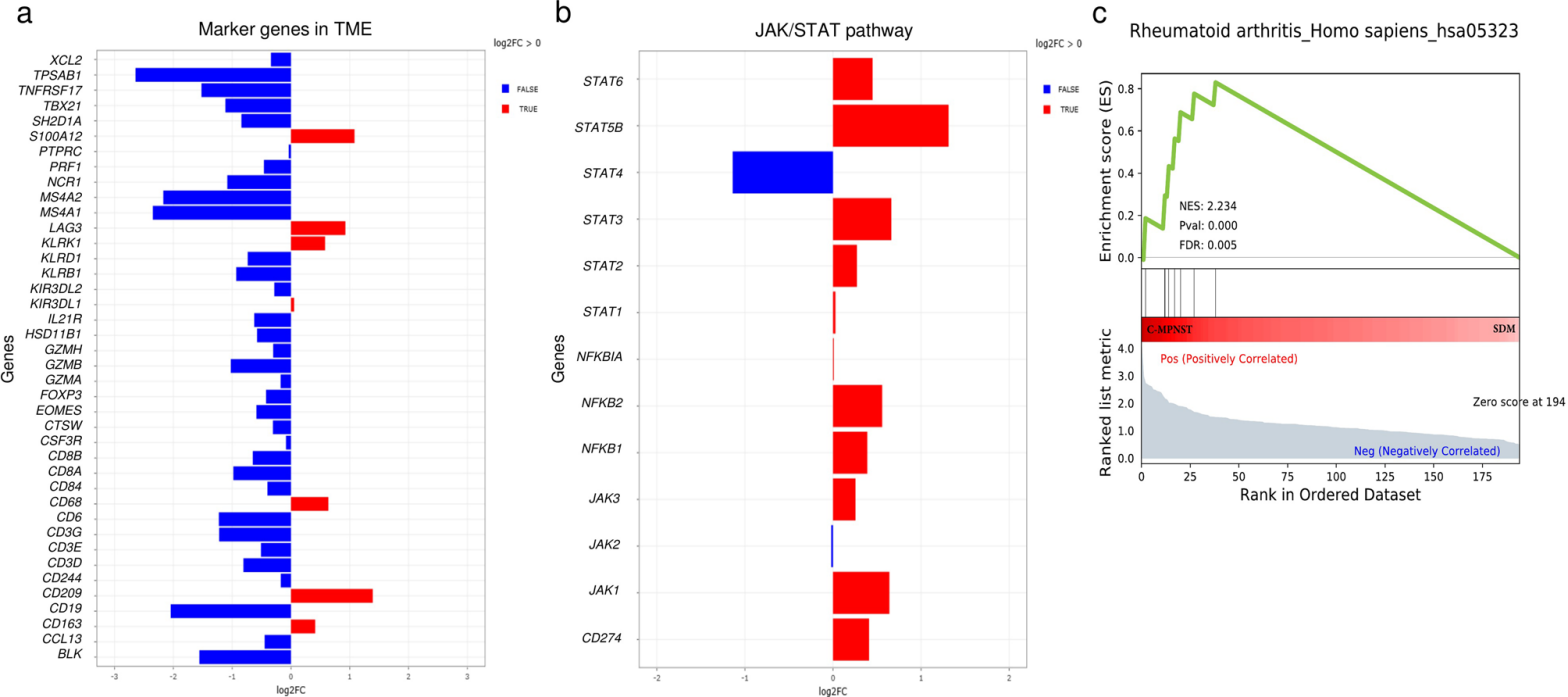
Comparison of differential gene expression (DGE) between C-MPNST and SCM. (**a**) Adjusted DGE of 60 marker genes defining 14 cell types in the TME, and (**b**) JAK/STAT signaling pathway. DGE highlights important effectors/downstream signaling pathways in C-MPNSTs and SCMs. The values are represented in normalized log2FC ratios (cutoff > 0) with an adjusted FDR < 0.01. Red color denotes C-MPNST group and blue color the SCM group. (**c**) Corresponding GSEA analysis showing upregulation of “RA pathway” signatures in C-MPNST vs. SCM. ES, enrichment score; NES, normalized ES; FDR, false discovery rate.

To investigate potential immunomodulatory differences in the TME composition of C-MPNSTs and SCMs we employed multiplex gene expression analysis using a customized 770-gene panel that encompasses 24 different immune cell types, common checkpoint inhibitors, cancer/testis antigens, and genes related to the adaptive and innate immune response. Select markers related to various cell types were scrutinized to detect changes in gene expression (Table 1). Exploitation of 770 genes, including 60 validated marker genes defining 14 cell types, was applied for DGE and GSEA using the Nanostring platform. While both entities showed common activation of key pathways such as NF-κB and JAK/STAT pathways, important differences were noted at the gene level. DGE analysis of C-MPNST cases revealed overexpression of key genes corresponding to macrophages (*CD68*, *CD163*), exhausted CD8^+^ T cells (*LAG3*), and cytotoxic cells (*KLRK1*) (Fig. 2a). On the contrary, a predominance of mast cells was observed in the TME of SCMs as evidenced by overexpression of *MS4A (MS4A1* and *MS4A2)* in the 60 marker genes (Fig. 2a) and *STAT4* in the JAK/STAT pathway (Fig. 2b). Of note, gene transcripts associated with activation of M1 macrophages (*STAT1*, *STAT2*), M2 macrophages (*JAK1*, *JAK3*) and *CD274* were overexpressed in C-MPNSTs (Fig. 2b). These findings are concordant to the perturbed pathways identified in a previous study using network analysis^11^.

**Table 1:** Summary of the marker genes and immune cells in the TME.

Cell type	Selected marker genes
B cells	*BLK*, *CD19*, *FCRL2*, *MS4A1*, *KIAA0125*, *TNFRSF17*, *TCL1A*, *SPIB*, *PNOC*
CD45^+^ cells	*PTRPC*
Cytotoxic cells	*PRF1*, *GZMA*, *GZMB*, *NKG7*, *GZMH*, *KLRK1*
Dendritic cells	*KLRB1*, *KLRD1*, *CTSW*, *GNLY*, *CCL13*, *CD209*, *HSD11B1*
Exhausted CD8^+^ cells	*LAG3*, *CD244*, *EOMES*, *PTGER4*
Macrophages	*CD68*, *CD84*, *CD163*, *MS4A4A*
Mast cells	*TPSB2*, *TPCAB1*, *CPA3*, *MS4A2*, *HDC*
Neutrophils	*FPR1*, *SIGLEC5*, *CSF3R*, *FCAR*, *FCGR3B*, *CEACAM3*, *S100A12*
NK CD56^dim^ cells	*KIR2DL3*, *KIR2DL1*, *KIR2DL2*, *IL21R*
NK cells	*XCL1*, *XCL2*, *NCR1*
T cells	*CD6*, *CD3D*, *CD3E*, *SH3E*, *SH2D1A*, *TRAT1*, *CD3G*
Th1 cells	*TBX21*
Tregs	*FOXP3*
CD8^+^ T cells	*CD8A*, *CD8B*

*NK* natural killer; *Th1* type 1T helper; *Tregs* regulatory T cells.

Following identification of disparate immune signatures encountered in these two entities, we subsequently carried out GSEA to elucidate affected cancer immune pathways. Application of GSEA identified a significant upregulation of the “Rheumatoid arthritis” gene set in C-MPNST (Fig. 2c). Our analysis showed strong enrichment in specific genes related to pivotal growth factors (*TGFB2*, *VEGFA*), pro-inflammatory cytokines (*CXCL5*, *CXCL6*), lysosomal endopeptidases (*CTSL*), and specific human leukocyte antigen (HLA) alleles (*HLA-DQA1*, *HLA-DQB1*) in the C-MPNST group which have been reported to be intricately involved in the pathogenesis of rheumatoid arthritis. These pronounced differences in cellular composition provide evidence that the immune profiles of the TME in C-MPNSTs and SCMs are different.

### Genome-wide methylation profiling reveals distinct genomic aberrations correlating gene expression and TME composition

**Figure 3:**
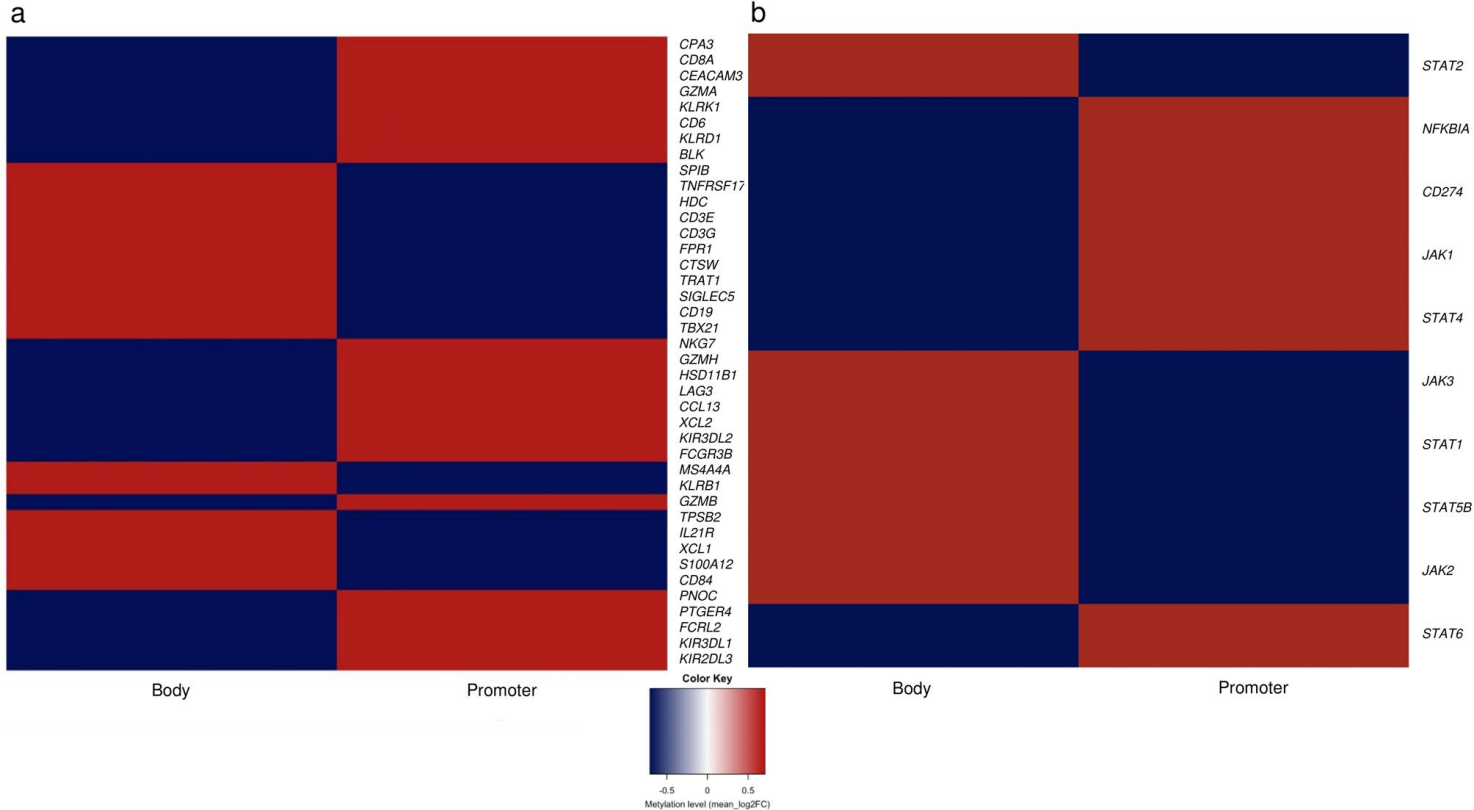
Evaluation of genome wide methylation levels between C-MPNST and SCM. (**a**) Differential methylation heatmap of the 60 above-mentioned genes and the (**b**) JAK/STAT pathway genes. Y-axis and x-axis represent the genes and gene region (body vs. promoter), respectively. The methylation status is expressed through the normalized *β* score with a range from − 0.5 (hypomethylated/blue) to + 0.5 (hypermethylated/red).

In order to investigate potential epigenetic events underlying the expression of the 60 above-mentioned genes, as well as those implicated in the JAK/STAT pathway, we applied an Infinium Methylation EPIC array targeting 866,562 methylation site (CpG) islands to assess genome-wide methylation levels. As expected, a positive correlation was seen between promoter hypomethylation and expression levels of genes catalogued among the 60 marker gene set and the JAK/STAT pathway. We further explored the methylation status of both promoter and body regions of the different genes of interest within this gene set. Surprisingly, key overexpressed genes from both the 60 above-mentioned gene set (*LAG3* and *CPA3*) and from the JAK/STAT pathway (*STAT4, JAK1*, *CD274*) showed hypermethylation of the promoters and hypomethylation of gene bodies as highlighted by the color coded heatmap (Fig. 3a,b). We then assessed the methylation status of gene bodies and expression of genes involved in the JAK/STAT pathway and found a positive correlation (Pearson correlation coefficient: *r* = 0.37; *p* = 0.01). This observation raised the possibility whether expression of these genes is not regulated via the promoter regions but through epigenetic mechanisms pertaining to gene bodies.

**Figure 4:**
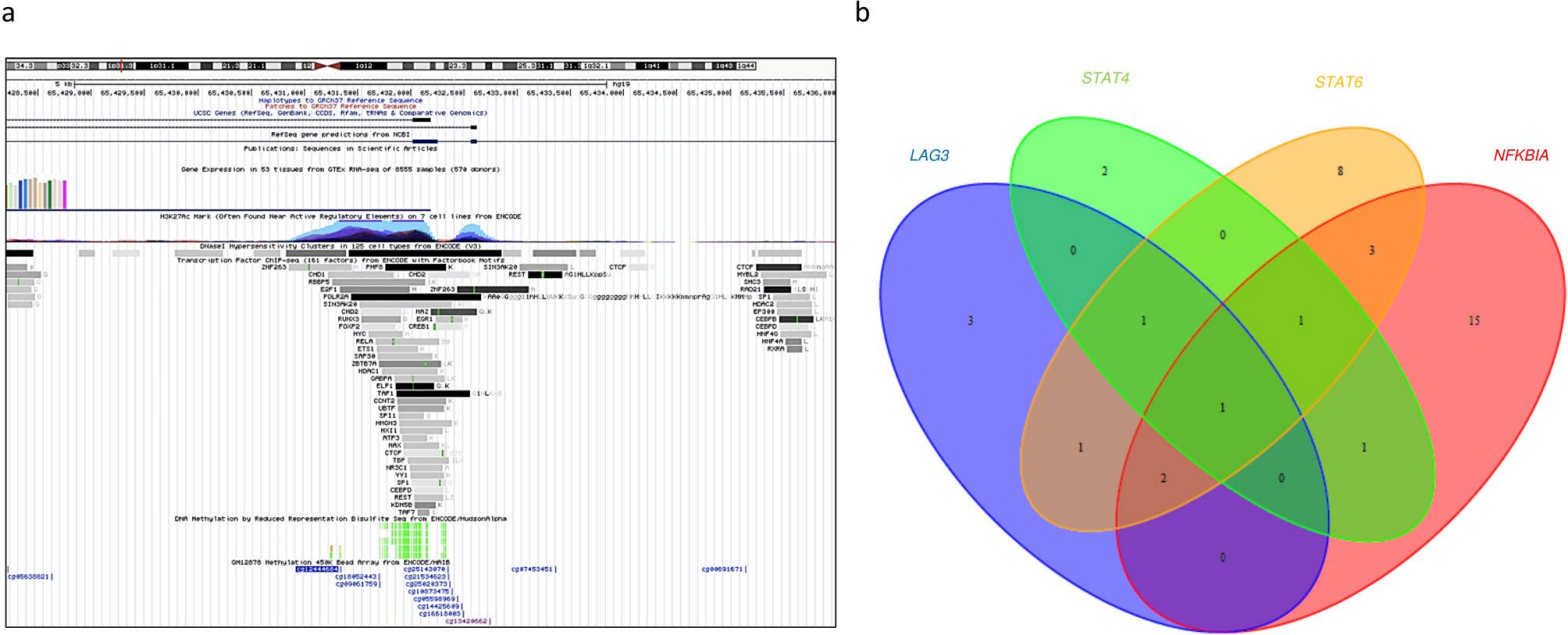
ENCODE data demonstrating *CTCF* as the common transcription factor in the study set. (**a**) Overlay of the hypomethylated body probes identified enriched peaks from the ENCODE ChIP-seq data corresponding to transcription factor/enhancer binding sites (also referred to as H3K27Ac histone mark) including *TAF1, POLR2A* and *CTCF*. The levels of enrichment are color coded; light grey—low, and black—high levels of enrichment. The screenshot is derived from the UCSC Genome Browser (https://genome.ucsc.edu/). (**b**) Venn diagram showing the common transcription factors across different overexpressed genes with body hypomethylation showing *CTCF* as the common denominator.

To gain further mechanistic insight, we mapped the hypomethylated probe sequences to the Encyclopedia of DNA Elements (ENCODE) consortium data set to investigate protein-chromatin interactions from the transcription factor ChIP-sequencing (TF ChIP-seq) pipeline. Analysis of publically available data revealed binding sites for numerous transcription factors such as *TAF1, POLR2A, ZNF263* and *CTCF*. Comparison of these gene lists using Venn diagrams identified *CTCF* as the common transcription factor at the site of the hypomethylated probe for all the above-mentioned overexpressed genes (Fig. 4a,b). These findings suggest an important role for *CTCF* in regulating expression of genes through gene bodies.

### Epigenetic deconvolution of methylation data corroborates TME differences identified on DGE analysis

**Figure 5:**
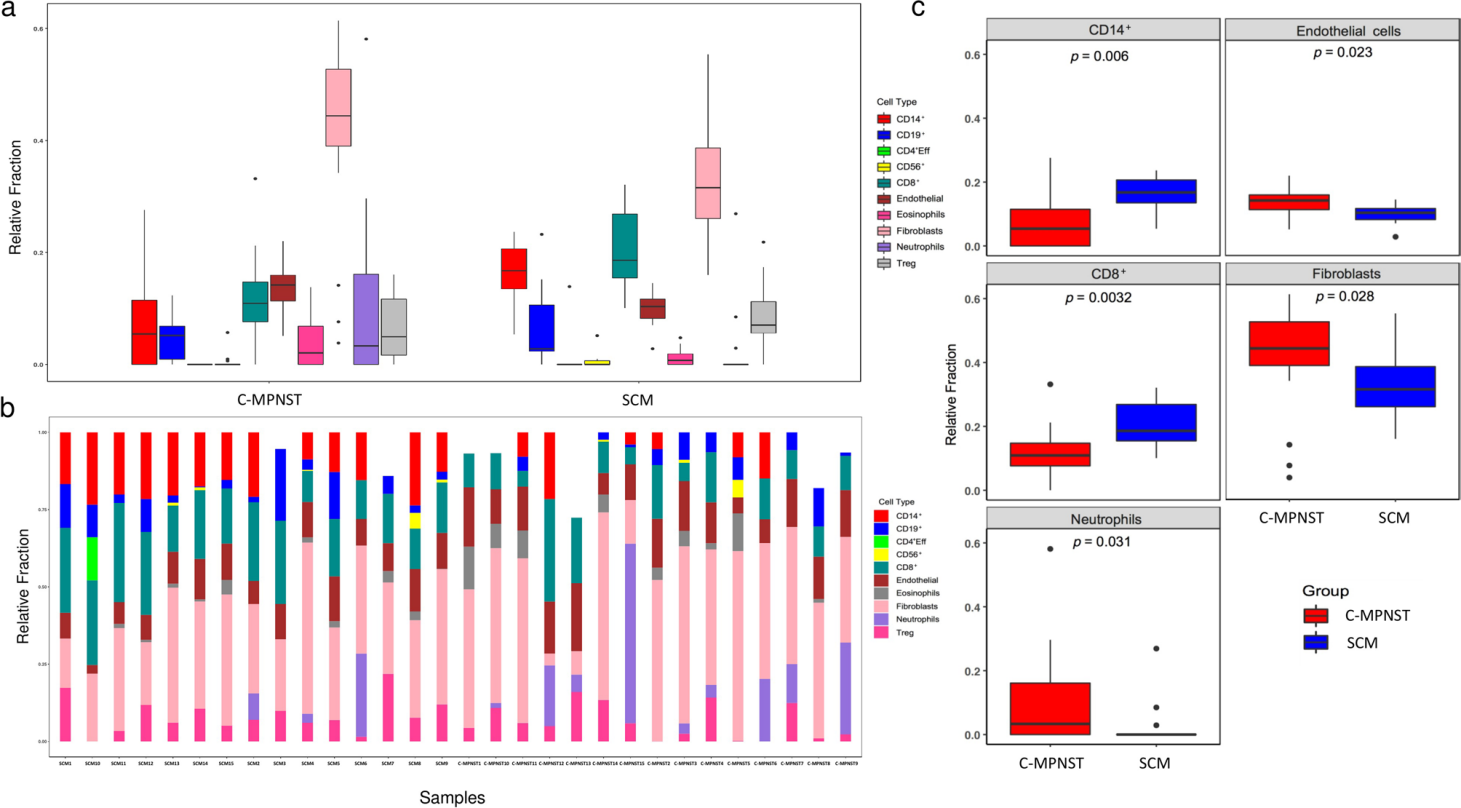
MethylCIBERSORT analysis was used to determine the TME composition across C-MPNST and SCM. (**a**) Color coded box plot representation of the different cell populations showing significant difference with middle line = median value; lower line = lower 25th percentile; upper line = upper 75th percentile; vertical line—confidence interval and points as outlier values. Note the significant enrichment (*p* < 0.05; Kruskal–Wallis test for comparison). (**b**) Summary of the different TME composition in each of the studied cases in the cohort. The x-axis is for the case number; y-axis  is the relative ratio of the specific color-coded population to the overall TME populations using MethylCIBERSORT. (**c**) Representation of the statistically significant populations enriched in the C-MPNST and SCM groups.

Using MethylCIBERSORT we extended our analysis and identified marked alterations between the TME composition of C-MPNSTs and SCMs (Fig. 5a,b). In C-MPNSTs, MethylCIBERSORT detected a predominance of endothelial cells (*p* = 0.023, Kruskal–Wallis test), neutrophils (*p* = 0.031, Kruskal–Wallis test), and cancer-associated fibroblasts in the TME (*p* = 0.028, Kruskal–Wallis test), while a strong enrichment for CD8^+^ T cells (*p* = 0.0032, Kruskal–Wallis test) and CD14^+^ cells (*p* = 0.006, Kruskal–Wallis test) was seen in the SCM group set (Fig. 5c). No statistically significant differences were found in CD19^+^ B cells (*p* = 0.6, Kruskal–Wallis test), CD4^+^ effector T cells (*p* = 0.35, Kruskal–Wallis test), CD56^+^ natural killer cells (*p* = 0.49, Kruskal–Wallis test), eosinophils (*p* = 0.29, Kruskal–Wallis test) and regulatory T cells (*p* = 0.25, Kruskal–Wallis test). These observations further validate the abundance of specific cell types within the TME of C-MPNSTs and SCMs, and substantiate the associations encountered utilizing DGE analysis.

## Discussion

In the current study, we pursued to explore the epigenetic events that could potentially explain the TME composition and differences in the distribution of various cell types among C-MPNSTs and SCMs to identify targets that would merit further investigation in future studies. Application of DGE analysis revealed distinct cell infiltration signatures within the TME of C-MPNSTs and SCMs. Results showed marked enrichment in exhausted CD8^+^ T cells and macrophages within the TME of C-MPNSTs as supported by *LAG3, CD68* and *CD163* overexpression, in contrast to a predominance of mast cells encountered in the TME of SCMs with overexpression of *MS4A (MS4A1* and *MS4A2), CPA3* and *STAT4*. Furthermore, GSEA identified a significant upregulation of the “Rheumatoid arthritis” gene set in C-MPNSTs. Interestingly, we observed promoter hypermethylation and gene body hypomethylation in a subset of overexpressed genes, including *STAT4*, *LAG3*, *CPA3*, *CD274* and *JAK1*. These findings suggest a potential role implicating gene bodies as regulators for expression of these respective genes.

DNA methylation plays pivotal roles in governing cellular processes such as regulation of gene transcription, chromatin structure remodeling, stability, tissue differentiation and X-chromosome inactivation^13–16^. The functionality of promoter hypermethylation has been well investigated in numerous studies across multiple decades, however the precise role of methylated gene bodies is poorly understood to date. Promoter hypermethylation results in the transcriptional silencing of genes, and aberrant methylation is now regarded as one of the hallmarks of cancer^13,17^. As such, investigators have pursued to mechanistically study whether a causal relationship exists between gene body methylation and gene expression^18–21^.

Chromatin structure and spatial positioning of the genome both have crucial implications on genome function^22^. CCCTC-Binding Factor (CTCF) is an essential DNA-binding protein involved in diverse functions such as mediating functional intra- and interchromosomal interactions to maintain genomic integrity, and restriction of the *CTCF* binding interface can result in alterations in gene expression^23,24^. Mechanistically, hypermethylation of genome *CTCF* binding sites or microdeletions present within these sites have been implicated in tumorigenesis and induction of oncogene expression^25,26^. *CTCF* has also been reported to pause RNA polymerase II, which in turn may have repercussions on splicing^27^. Our study represents an example of a possible link between gene body methylation status and gene expression, while additionally supporting the disparate nature of the TME among C-MPNSTs and SCMs as shown by DGE and further corroborated by the MethylCIBERSORT results. Interrogation of overexpressed genes *STAT4*, *CD274, JAK1, LAG3* and *CPA3* revealed hypermethylated promoter regions and hypomethylated gene bodies, implying possible roles for gene bodies in gene expression. This was further supported by the fact that overlay with ChIP-seq analysis identified important binding sites on these hypomethylated probe sequences to pivotal transcription factor *CTCF*. Our analysis suggests that *CTCF* may represent a shared central player in the activation of the JAK/STAT pathway involved in tumorigenesis through differential expression of specific genes in both studied entities (C-MPNST and SCM). In fact, *CTCF* gene downregulation was shown to suppress cell proliferation, cell invasion and facilitate cell apoptosis in prostate cancer cell lines^28^. Whether inhibition of *CTCF* would have a similar effect in C-MPNST and SCM certainly warrants further investigation as it could represent an additional therapeutic modality to commonly used targeted therapies in C-MPNST and immunotherapy in SCM.

Interrogation of C-MPNSTs in our cohort revealed a preponderance of neutrophils, cancer-associated fibroblasts and endothelial cells. The intricate and dynamic disposition of the TME and signaling networks within this framework may play substantial roles in regulating responses to immunotherapeutic modalities. Neutrophils are highly abundant in the TME and studies have shown that the presence of a neutrophil-related gene signature has been associated with dismal prognosis across 39 types of cancer, whereas additional findings in mouse models have linked the presence of neutrophils to higher rates of metastatic tumor progression^29–32^. Moreover, cancer-associated fibroblasts are implicated in facilitating tumor progression and metastasis via cytokine secretion and epithelial-to-mesenchymal transition^33^. These lines of evidence suggest that the relative abundance of neutrophils and cancer-associated fibroblasts could potentially explain the more aggressive behavior and lack of immunotherapeutic response of C-MPNSTs in comparison to SCMs.

Infiltration of CD8^+^ T cells within tumor tissue is required to mediate cytotoxic effects against tumor cells. Tumor infiltrating lymphocytes can exert immunologic responses, whereas tumor regression has been shown in patients with metastatic melanoma following adoptive T cell transfer^34,35^. The predominance of CD8^+^ T cells encountered in SCMs, designating them as ‘hot tumors’, could contribute to reported immunotherapeutic responses in these tumors notably with FDA approved therapies such as pembrolizumab or nivolumab.

In conclusion, our findings demonstrate that the TME in two morphologically overlapping entities is regulated by distinct epigenetic mechanisms which could explain the difference in clinical outcomes and prognosis between them. Furthermore, our deconvolution of the TME composition highlights specific cell populations such as neutrophils that warrants further investigation as potential targets for novel selective neutrophil exocytosis inhibitors (Nexinhibs)^36^ in addition to currently available targeted therapies in the C-MPNST group. Finally, based on the identification of *CTCF* as a common underlying epigenetic modulator for different gene expression profiles in both entities, a centered therapeutic approach focusing on this particular transcription factor using selective *CTCF* inhibitors merits further investigation.

## Methods

### Case selection

After institutional review board (IRB) approval at the 2 collaborating institutions, thirty cases including 15 cases of C-MPNSTs and 15 cases of SCMs were selected from patients diagnosed during the period from 2000 through 2015. All available slides for each case were reviewed by two pathologists certified in dermatopathology and bone and soft tissue pathology in order to confirm the diagnoses and select the most viable tumor areas for DNA and RNA extraction. All SCM cases were from resected tumors with an intra-epidermal in situ component with S100 (diffuse and strong nuclear and cytoplasmic), nestin, and SOX10 immunopositivity (all staining performed as part of the initial clinical workup), and moderate-to-severe solar elastosis. Criteria for diagnosing C-MPNST included the following: (1) lack of intra-epidermal in situ component and only dermal/subcutaneous involvement; (2) lack of moderate-to-severe solar elastosis; (3) patchy weak-to-moderate nuclear and cytoplasmic S100 immunopositivity; and (4) the presence of a remnant neurofibroma from which the tumor arose (whenever applicable). In addition, any lesion exhibiting severe cytologic atypia with geographic necrosis and brisk mitotic activity was considered a C-MPNST. Using the following system, all cases had sufficient histologic and immunohistochemical findings that enabled an unequivocal diagnosis.

### Tissue selection, nucleic acid isolation, DNA and RNA processing

For each specimen, ten 10 μm-thick formalin-fixed, paraffin-embedded (FFPE) sections were cut from a single representative block. Macrodissection was performed with a scalpel and focused on the areas of greatest tumor cell density to try to ensure that tumor cells accounted for at least 70% of the material analyzed for each case. DNA was recovered using the PicoPure DNA extraction kit (Thermo Fisher, Life Technologies), which enables recovery of genomic DNA from FFPE tissue^11^. DNA was then subjected to bead purification with the Sphere quality control kit (Thermo Fisher, Life Technologies). DNA was quantified using the Qubit 2.0 fluorometer (Life Technologies). The DNA was bisulfite converted using the EZ-96 DNA methylation kit (Zymo Research, Irvine, CA). Extracted DNA was restored using the Infinium HD FFPE DNA restore kit (Illumina, San Diego, CA) prior to hybridization on the bead chips provided by the manufacturer (Illumina). Quality metrics for extracted RNA included both quantitative and qualitative parameters. RNA quantification was performed with the Qubit 2.0 Fluorometer (Life Technologies). Qualitative RNA integrity was performed with the Agilent 2100 Bioanalyzer System (Agilent software, Santa Clara, CA, USA) and corresponding RNA kits. Electropherograms showing fragment distribution were reviewed; only cases with an RNA integrity number of > 3 were selected.

### Gene expression analysis

Multiplex gene expression analysis with 770 genes from 24 different immune cell types, common checkpoint inhibitors, cancer/testis antigens, and genes covering both the adaptive and innate immune response was performed using extracted RNA from patients' samples as previously described^11^. Differential gene expression (DGE) (log2 FC) and gene set enrichment analysis (GSEA) of 770 genes including 60 validated marker genes, defining 14 cell types in the tumor microenvironment, was performed using the Nanostring platform (Table 1). The set of 60 genes was then validated against immunohistochemistry and flow cytometry in immune cells from tumor microenvironment across 23 types of TCGA solid tumors. DGE and GSEA, correlation between gene expression and methylation status of promoter and body regions was performed using R project with *p* < 0.05 and FDR < 0.01.

### Methylation analysis

The Infinium Methylation EPIC array (Illumina) was used to determine the DNA methylation status of 866,562 CpG sites, following the manufacturer’s instructions. Minfi R package was used to process and analyze the methylation data^37^. Using minfi, the probes were quantile normalized and background adjusted. The resulting set of samples and probes was used for differentially methylated probes analysis. The differentially methylated probes finder function in minfi was used to identify differentially methylated probes for the 60 validated marker genes of interest in two comparisons: (1) SCM vs. C-MPNST, and (2) C-MPNST vs. SCM. Probes with statistical significance using Benjamini–Hochberg false discovery rate *q* cutoff of *q* < 0.05 were considered most significant, and corresponding heatmaps are shown. *β*-values for all 866,562 CpG sites tested were defined as the ratio of fluorescence intensity of the methylated probe over the overall intensity of probes. *β* < 0.2 indicated hypomethylation (blue); *β* > 0.8 indicated hypermethylation (red).

For TME cell population assessment we used MethylCIBERSORT^38^—an R package for deconvolution that helps in estimating the tumor purity and cellular composition from DNA methylation data. Raw idats were processed and beta values were obtained. Signature genes were generated as described previously^38^. Beta matrix for tumor samples were loaded into CIBERSORT software along with the signature genes and the samples were deconvoluted to get the accurate estimate of the immune cell populations^38^. All statistical analysis and modeling was performed using the open-source software R (https://www.r-project.org/). All graphs and heatmaps were generated using the R package. Unsupervised hierarchical clustering was done with Euclidean measure for distance matrix and complete agglomeration method for clustering was used for unsupervised hierarchical clustering.

### Ethics statement

Our research was conducted after approval by the ethics committee of the Office of Science and Research at NYU Langone Health (Study # i18-00637; Correspondence signed by director of the IRB: Helen Panageas) and followed the guidelines imposed by our institution. All methods listed in our research follow our institutional guidelines. A waiver of authorization for consent was approved by the review committee as there was no direct interaction with the patients and the retrospective nature of the study. The study involved no more than minimal risk to the privacy of our patients. All patients were de-identified and assigned a study number. The de-identified data will be stored on the REDCAP database which is HIPPA compliant and only accessed by the first and last investigators listed in the manuscript.
